# Half-Sandwich Ru(II) Halogenido, Valproato and 4-Phenylbutyrato Complexes Containing 2,2′-Dipyridylamine: Synthesis, Characterization, Solution Chemistry and In Vitro Cytotoxicity

**DOI:** 10.3390/molecules21121725

**Published:** 2016-12-15

**Authors:** Pavel Štarha, Zdeněk Trávníček, Radka Křikavová, Zdeněk Dvořák

**Affiliations:** 1 Department of Inorganic Chemistry & Regional Centre of Advanced Technologies and Materials, Faculty of Science, Palacký University in Olomouc, 17. listopadu 12, 771 46 Olomouc, Czech Republic; pavel.starha@upol.cz (P.S.); radka.krikavova@upol.cz (R.K.); 2 Department of Cell Biology and Genetics & Regional Centre of Advanced Technologies and Materials, Faculty of Science, Palacký University in Olomouc, Šlechtitelů 27, 783 71 Olomouc, Czech Republic; zdenek.dvorak@upol.cz

**Keywords:** ruthenium, half-sandwich, 2,2′-dipyridylamine, X-ray structure, solution behaviour, in vitro cytotoxicity

## Abstract

Halogenido and carboxylato Ru(II) half-sandwich complexes of the general composition [Ru(η^6^-*p*-cym)(dpa)X]PF_6_ (**1**–**5**) were prepared and thoroughly characterized with various techniques (e.g., mass spectrometry, NMR spectroscopy and X-ray analysis); dpa = 2,2′-dipyridylamine; *p*-cym = *p*-cymene; X = Cl^−^ (for **1**), Br^−^ (for **2**), I^−^ (for **3**), valproate(1−) (for **4**) or 4-phenylbutyrate(1−) (for **5**). A single-crystal X-ray analysis showed a pseudo-octahedral piano-stool geometry of [Ru(η^6^-*p*-cym)(dpa)I]PF_6_ (**3**), with a η^6^-coordinated *p*-cymene, bidentate *N*-donor dpa ligand and iodido ligand coordinated to the Ru(II) atom. The results of the ^1^H-NMR solution behaviour studies proved that the complexes **1**–**5** hydrolyse were in the mixture of solvents used (10% MeOD-*d*_4_/90% D_2_O). Complexes **1**–**5** were in vitro inactive against the A2780 human ovarian carcinoma cell line, up to the highest tested concentration (IC_50_ > 100 μM).

## 1. Introduction

Organometallic Ru(II) half-sandwich complexes represent one of the most promising group of newly developed substances for the treatment of cancer [1,2]. These complexes typically adopt a piano-stool geometry with η^6^-coordinated arene (e.g., *p*-cymene or benzene), chelating (XY) and monodentate (Z; typically chlorido ligand) ligands, together producing the compounds of the general composition [Ru^II^(η^6^-arene)(XY)Z]^0/+^. It has been reported that the in vitro cytotoxicity of these complexes can be improved when the chlorido ligand is replaced by different halogenido ligand, as exemplified on the [Ru(η^6^-*p*-cym)(L^1^)Cl]PF_6_ and [Ru(η^6^-*p*-cym)(L^1^)I]PF_6_ complexes, showing the IC_50_ values of 16.2, and 3.0 µM, respectively, against the A2780 human ovarian carcinoma cells; L^1^ = *N,N*-dimethyl-*N*′-(2-pyridinylmethylene) [3].

Quite recently, the simple organic valproato(1–) (VP) and 4-phenylbutyrato(1–) (PB) ligands, derived from the histone deacetylase inhibitors (HDACi) valproic acid (VPA) [4] and sodium 4-phenylbutyrate (Na(PB)) [5], have been used as the releasable *O*-donor ligands of the platinum(IV) complexes derived from the clinically used platinum(II) drugs *cisplatin* and *oxaliplatin*. Remarkably, the studied platinum(IV) valproato and 4-phenylbutyrato complexes exhibited significantly higher in vitro cytotoxicity than their hydroxidoplatinum(IV) analogues [6,7,8,9]. For example, the complex *trans*-[Pt(dach)(ox)(VP)_2_] (IC_50_ = 1.3 µM) significantly exceeded potency of *trans*-[Pt(dach)(OH)_2_(ox)] (IC_50_ = 11.4 µM) against the A2780 cells [8]; dach = 1,2-diaminocyclohexane, ox = oxalate(2–).

**Figure 1:**
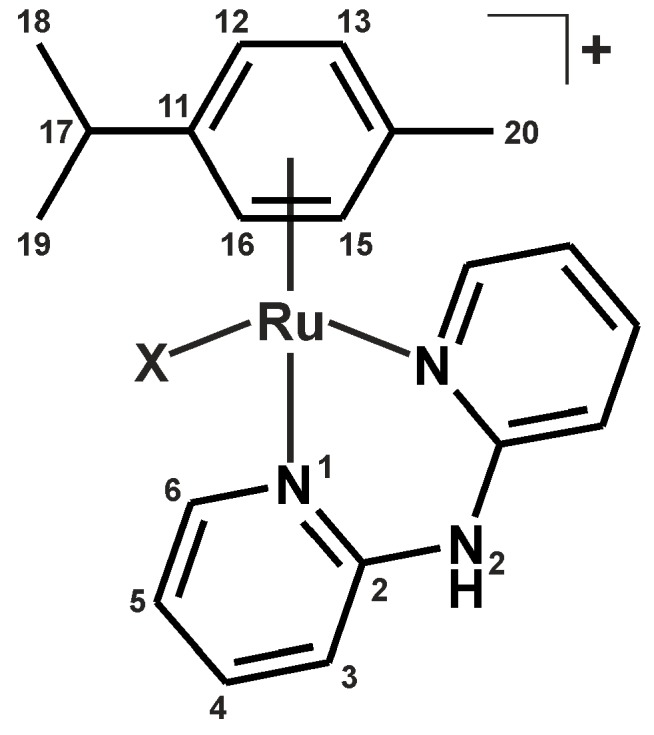
General structural formula of the studied complexes **1**–**5** given with the atom numbering scheme for the *p*-cym and dpa ligands; *p*-cym = *p*-cymene; dpa = 2,2′-dipyridylamine; X = Cl^−^ (**1**), Br^−^ (**2**), I^−^ (**3**), valproate(1−) (**4**) or 4-phenylbutyrate(1−) (**5**).

In this work, we decided to combine the above-described approaches, and study the effect of the replacement of the chlorido ligand with other halogenido ligands (a known approach utilized, for example, in the reference [3]) as well as by the VP and PB ligands (innovative approach in the field of Ru(II) anticancer complexes) on the in vitro cytotoxicity against the A2780 cells. For this purpose, we chose the easily-obtainable [Ru(η^6^-*p*-cym)(dpa)Cl]PF_6_ complex (Figure 1) [10], recently reported as having moderate, and thus possibly tunable, in vitro cytotoxicity against the MCF-7 cancer cells (IC_50_ = 40.8 µM) [11]. From the coordination chemistry point of view, this is the first report on Ru(II) complexes containing the VP ligand. Regarding the ruthenium complexes containing the PB ligand, they have been used as either bidentate *O*,*O*-coordinated ligand in the [Ru(µ-PB)(CO)_2_(L^2^)]_2_ complex, or as η^6^-coordinated 4-phenylbutyric acid (PBA) in the [Ru(η^6^-PBA)(L^3^)Cl]^+^ or [Ru(η^6^-ar)(η^6^-PBA)]^2+^ complexes; L^2^ = pyridine or triphenylphosphane, L^3^ = e.g., 1,10-phenanthroline or benzaldehyde monothiosemicarbazone, and ar = e.g., 1,2,3,4,5,6-hexamethylbenzene [12,13,14]. However, to the best of our knowledge, neither VP nor PB ligand has been reported in the literature to date as the monodentate *O*-donor ligand of any ruthenium complex.

## 2. Results and Discussion

### 2.1. Synthesis and General Properties

Recently, the preparation [10,11,15,16], X-ray structure [10] and in vitro cytotoxicity [11] of the simple ruthenium(II) complex [Ru(η^6^-*p*-cym)(dpa)Cl]PF_6_ were reported. In this work, its structure was modified by either different halogenido ligands (bromido or iodido) or monodentate *O*-coordinated carboxylato ligands (valproato or 4-phenylbutyrato). The obtained products were thoroughly characterized, their solution chemistry was studied by ^1^H-NMR and their in vitro cytotoxicity against the A2780 cells was assessed.

The synthesis of the herein used complex [Ru(η^6^-*p*-cym)(dpa)Cl]^+^ was reported in the literature, but with different reaction conditions (4 h/ambient temperature [15], 4 h/reflux [11], 6 h/reflux [16] or overnight stirring/50 °C [10]). In this work, the reaction time was considerably shortened to only 1 min, by using a microwave reactor. For the bromido (**2**) and iodido (**3**) complexes, instead of the known protocol starting from the appropriate dimeric compounds, [Ru(µ-Br)(η^6^-*p*-cym)Br]_2_ and [Ru(µ-I)(η^6^-*p*-cym)I]_2_ [17], the easily-obtainable chloride salt [Ru(η^6^-*p*-cym)(dpa)Cl]Cl (1*) was dechlorinated by 2 molar equivalents of silver triflate, followed by the addition of the appropriate potassium halogenide to form [Ru(η^6^-*p*-cym)(dpa)X]^+^ (**2**, **3**). In the case of carboxylato complexes **4** and **5**, a one-step replacement of the chlorido ligand of **1** by the VP or PB ones was achieved using the silver carboxylates Ag(VP) (for **4**) or Ag(PB) (for **5**).

**Figure 2:**
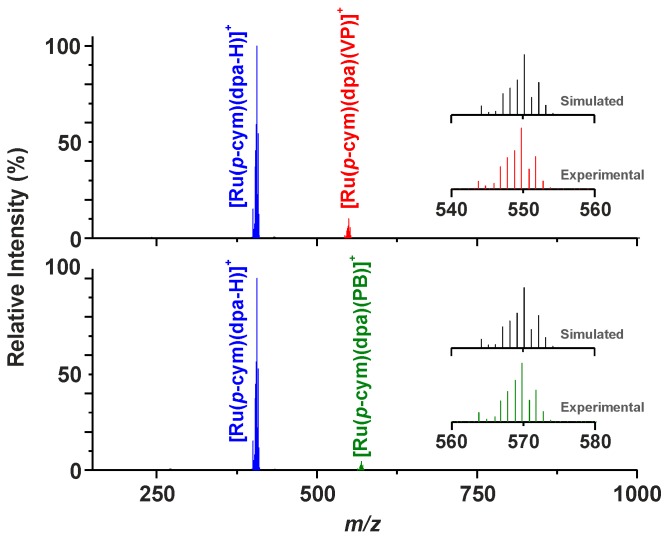
ESI+ mass spectra of the carboxylato complexes [Ru(η^6^*-p*-cym)(dpa)(VP)]PF_6_ (**4**; **top**) and [Ru(η^6^*-p*-cym)(dpa)(PB)]PF_6_ (**5**; **bottom**), given with a comparison of the experimental and simulated isotopic distributions of the [Ru(*p*-cym)(dpa)(VP)]^+^ and [Ru(*p*-cym)(dpa)(PB)]^+^ species (**insets**).

The prepared complexes of the general formula [Ru(η^6^-*p*-cym)(dpa)X]PF_6_ (Figure 1) were thoroughly characterized, using elemental analysis, RP-HPLC, FTIR spectroscopy, ESI+ mass spectrometry, ^1^H-NMR and ^13^C-NMR spectroscopy, and a single crystal X-ray analysis. The composition of the complexes **1**–**5** was proved by the results of elemental analysis, with up to 0.4% differences between the calculated and found contents of C, H, and N. The RP-HPLC experiments proved >99% purity of the complexes **1**–**5**. FTIR spectra of the complexes **1**–**5** contained the characteristic peaks of ν(C–H)_aliph_ (2930–2980 cm^−1^) of *p*-cymene, as well as ν(C–H)_ar_ (3000–3200 cm^−1^), and ν(C–C)_ar_ and ν(C–N)_ar_ (1435, 1470 and 1580 cm^−1^) of the dpa and *p*-cymene ligands [18]. The maxima detected at ca. 830 cm^−1^ belong to the ν(P–F) vibrations [19]. The peaks assignable to the characteristic vibrations of the VP (e.g., 2930, 1640 or 1380 cm^−1^) and PB (e.g., 2970, 1640, 1385 or 1300 cm^−1^) ligands [20,21] clearly showed in the FTIR spectra of the complexes **4** and **5**. The peaks whose mass corresponded to the complex cations, i.e., [Ru(*p*-cym)(dpa)X]^+^, of the studied complexes **1**–**5** were detected in the ESI+ mass spectra (Figure 2). A release of the halogenido (for **1**–**3**) or carboxylato (for **4** and **5**) ligands under the used electrospray ionization conditions led to the [Ru(*p*-cym)(dpa–H)]^+^ species, whose peaks appeared at 406.2 *m*/*z* uniformly for all complexes **1**–**5**.

**Figure 3:**
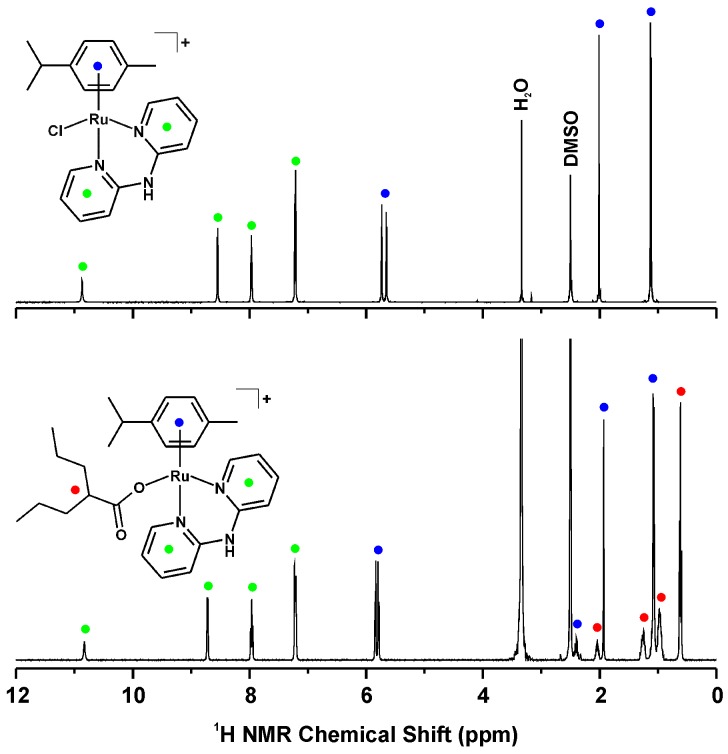
^1^H-NMR spectra of [Ru(η^6^-*p*-cym)(dpa)Cl]PF_6_ (**1**; **top**) and [Ru(η^6^-*p*-cym)(dpa)VP]PF_6_ (**4**; **bottom**), given together with the general assignment of the observed signals, as follows; green dots for the dpa signals, blue dots for the *p*-cym signals and red dots for the VP signals.

The ^1^H- and ^13^C-NMR spectra of the complexes **1**–**5** (measured in DMSO-*d_6_*) contained all the signals of both the *p*-cym and dpa ligands (Figure 3), and only the N2–H signal was not detected in the ^1^H-NMR spectrum of complex **5**. The septet of C17–H (i.e., H_3_C–C*H*–CH_3_) was overlapped by the signal of the used solvent for complexes **1**–**3**, but this signal was clearly detected in the 2D NMR spectra, as well as in the ^1^H-NMR spectra obtained in different solvents (e.g., MeOD-*d*_4_). The signals of C4–H, C5–H and C6–H shifted downfield as a consequence of the coordination of the dpa ligand to the Ru(II) atom, while the signals detected for C3–H showed strong upfield shifts (Table 1).

The spectra obtained for the carboxylato complexes **4** and **5** contained, along with the signals of the *p*-cym and dpa ligands, the signals of the appropriate carboxylato ligands (Figure 3). The ^13^C-NMR signals of the carboxyl carbon atom (i.e., C21) were detected at ca. 181 ppm (for the VP-containing complex **4**) and 179 ppm (for the PB-containing complex **5**), and they were shifted by 4.0 ppm, and 4.7 ppm, respectively, as compared with the free carboxylic acids (i.e., VPA and PBA). The positions of these characteristic ^13^C-NMR signals of the complexes **4** and **5** were consistent with those reported for *trans*-[Pt(NH_3_)(py)(VP)_2_] (182.1 ppm), containing valproate(1−) as the monodentate *O*-donor ligand [22]. On the other hand, the formerly reported complexes [Ru(µ-PB)(CO)_2_(L^2^)]_2_, containing PB as the *O*-donor bridging ligand, and [Ru(η^6^-PBA)(L^3^)Cl]^+^ or [Ru(η^6^-ar)(η^6^-PBA)]^2+^, containing PBA as η^6^-coordinated ligand, showed different ^13^C-NMR δ values of their COO^−^ signals. In particular, the δ_COO–_ value for [Ru(µ-PB)(CO)_2_(L^2^)]_2_ equals 186.5 ppm [12], while it equals 175.0 ppm for [Ru(η^6^-PBA)(L^3^)Cl]^+^ [14]. Czomplexes **1**–**5** are stable in the used solvent (DMSO-*d*_6_), because no changes were detected in their ^1^H and ^13^C-NMR spectra even after 48 h of standing at ambient temperature.

### 2.2. Single Crystal X-ray Analysis

The complex [Ru(η^6^-*p*-cym)(dpa)I]PF_6_ (**3**) was characterized by a single crystal X-ray analysis. The crystal data and structure refinement are given in Table 2, while the selected bond lengths and angles can be found in Table 3.

**Figure 4:**
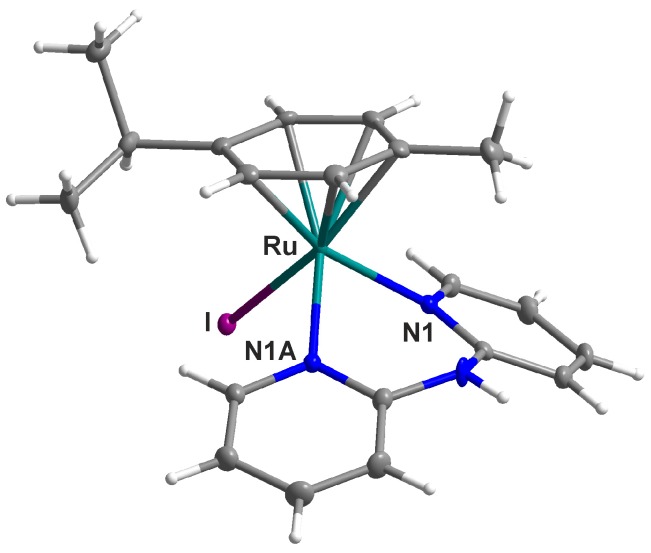
Molecular structure of [Ru(η^6^-*p*-cym)(dpa)I]PF_6_ (**3**). Non-hydrogen atoms are drawn as thermal ellipsoids at the 50% probability level. The PF_6_^−^ counterion has been omitted for clarity.

The complex **3** adopts a pseudo-octahedral piano-stool geometry (Figure 4), known to be a typical one for the half-sandwich Ru(II) complexes, including the recently reported complex [Ru(η^6^-*p*-cym)(dpa)Cl]PF_6_ (**1** in this work; [10]), whose structural parameters were included in Table 3 for comparative purposes. The Ru–N and Ru–*Cg* bond lengths are comparable for the chlorido (**1**; [10]) and iodido (**3**) complexes (Table 3); *Cg* = the centroid of the *p*-cymene aromatic ring. Moreover, the Ru–N bond lengths agreed well with the average value of 2.11(4) Å (the range of 2.025–2.219 Å) of 30 ruthenium complexes, such as [Ru(η^6^-bz)(dpa)Cl]PF_6_ [15], [Ru(η^6^-*p*-cym)(dpa)Cl]BF_4_ [16] or [Ru(η^6^-hmbz)(dpa)Cl]PF_6_∙CH_2_Cl_2_ [23], containing the *N*-donor dpa ligand, which have been deposited in the Cambridge Crystallographic Data Centre (CCDC) under the respective Cambridge Structural Database (CSD) refcodes; CSD version 5.37 updated to May 2016 [24]; bz = benzene, hmbz = 1,2,3,4,5,6-hexamethylbenzene.

To date, only three half-sandwich *p*-cymene-iodidoruthenium(II) complexes containing a bidentate *N*-donor ligand, namely [Ru(η^6^-*p*-cym)(L^4^)I]PF_6_ [25], [Ru(η^6^-*p*-cym)(en)I]I [26] and [Ru(η^6^-*p*-cym)(L^5^)I] [27], were crystallographically characterized; L^4^ = 2,2′-bipyrimidine, en = ethylene-1,2-diamine, HL^5^ = *N*-[(*1R,2R*)-2-(amino)-1,2-diphenylethyl]-4-methylbenzenesulfonamide. The average Ru–I bond length of these three complexes is 2.74(3) Å. However, only [Ru(η^6^-*p*-cym)(L^4^)I]PF_6_ is structurally similar to complex **3** with a heterocyclic chelating ligand coordinated to the Ru(II) atom through the two nitrogen atoms [25].

Both the aromatic rings of the dpa ligand form the dihedral angle of 37.00(8)°, while the dihedral angles between theses rings and *p*-cymene ring equal 29.46(8)° (formed by the ring containing the N1 atom) and 27.40(8)° (formed by the ring containing the N1A atom). The crystal structure of the complex **3** is stabilized by a variety of non-covalent contacts of the N–H···F and C–H···F types (Supplementary Materials Table S1 and Figure S1).

### 2.3. ^1^H-NMR Studies of Solution Chemistry and Interactions with Reduced Glutathione

As it is known for the anticancer complexes of various transition metals (e.g., platinum(II) [28] or ruthenium(II) [29] complexes), hydrolysis of the M–Cl bond/s is an activation step of their action enabling the interaction with the target biomolecules including DNA. Moreover, it is necessary to ensure that the studied complexes do not decompose in water or water-containing solution mimicking physiological conditions, as recently described for similar half-sandwich Ru(II) complexes [30].

After dissolution of the halogenido complexes **1**–**3** in a mixture of 10% MeOD-*d*_4_/90% D_2_O, the new sets of ^1^H-NMR signals of both the dpa and *p*-cym ligand were detected (e.g., at 7.90 ppm for C4–H or at 2.00 ppm for C20–H in the case of the complex **1**). The formation of these new signals is most likely connected with the hydrolysis of the original complexes, showing in the formation of the [Ru(η^6^-*p*-cym)(dpa)(H_2_O)]^2+^ and/or [Ru(η^6^-*p*-cym)(dpa)(OH)]^+^ species [2]. The integral intensity ratios of the signals of the initial halogenido complexes (**1**–**3**) and their hydrolyzed forms observed after 48 h of standing at ambient temperature (Figure S2) equaled approximately 1:3 (for **1**), 2:3 (for **2**) and 3:2 (for **3**). In other words, 75% (**1**), 60% (**2**) and 40% (**3**) of the studied halogenido complexes hydrolyzed in the used mixture of solvents after 48 h of standing at ambient temperature. The evidence that the new ^1^H-NMR signals detected in the spectra of the complexes **1**–**3** dissolved in a mixture of 10% MeOD-*d*_4_/90% D_2_O belong to the hydrolyzed complexes was obtained by the addition of 2 molar equivalents of KCl (for **1**), KBr (for **2**) or KI (for **3**) to the appropriate equilibrated solutions, which overturned the hydrolysis progress, resulting in disappearance of the signals of hydrolysates after next 24 h of standing at ambient temperature (Figure S2).

**Figure 5:**
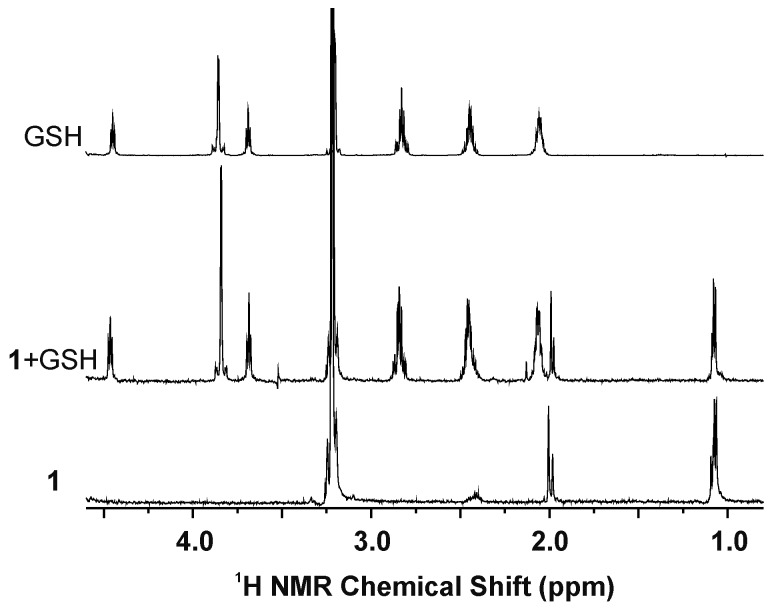
Parts of the ^1^H-NMR spectra of complex **1** and its mixture with 2 molar equivalents of the reduced glutathione (GSH), both after 48 h of standing at ambient temperature. ^1^H-NMR spectrum of free GSH is given for comparative purposes.

Glutathione (GSH) is a naturally occurring tripeptide (Glu–Cys–Gly), known to be responsible for the intracellular detoxification of various transition metals [31], including ruthenium [32]. Importantly, it has been reported that the decrease in the GSH level of cancer cells induced by co-application of a *γ*-glutamylcysteine synthetase inhibitor, l-buthionine sulfoximine (l-BSO), led to the cytotoxicity enhancement for similar half-sandwich Ru(II) complexes [3], thus indicating the important role of GSH for cytotoxicity of prospective anticancer ruthenium complexes. That is why similar experiments, as described above, were performed for complexes **1**–**5** dissolved in 10% MeOD-*d*_4_/90% D_2_O, with an addition of 2 molar equivalents of GSH. However, no evidence was obtained for the formation of the GS–Ru adducts of the studied halogenido complexes with GSH even after 48 h of standing at ambient temperature, because the ^1^H-NMR spectra of the mixtures of complexes **1**–**3** with GSH contained only the signals of the initial complexes (**1**–**3**), their hydrolysates (as described above) and those of free GSH (e.g., at 4.45 ppm and 2.85 ppm for Cys-α CH, and Cys-β CH_2_, respectively) (Figure 5).

**Figure 6:**
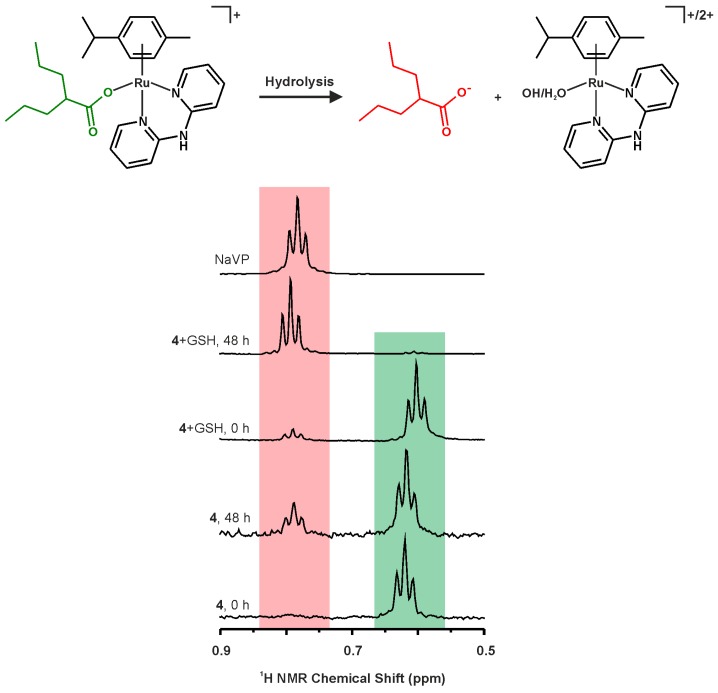
A schema of the hydrolysis of complex [Ru(η^6^-*p*-cym)(dpa)(VP)]PF_6_ (**4**) given together with the tentative compositions of the hydrolysates, i.e., the [Ru(η^6^-*p*-cym)(dpa)(H_2_O)]^2+^ and [Ru(η^6^-*p*-cym)(dpa)(OH)]^+^ species (**top**). ^1^H-NMR spectra of the representative signal of the terminal C25–H methyl group of released valproate(1–) anion (ca. 0.78 ppm; red) and the valproato ligand (ca. 0.62 ppm; green), as observed for complex **4**, without or with the addition of the reduced glutathione (GSH) at different time points. ^1^H-NMR spectrum of free sodium valproate (NaVP) is given for comparative purposes (**bottom**).

Regarding the carboxylato complexes **4** and **5**, their hydrolysis was connected with a release of the appropriate *O*-donor ligands, resulting in the complex species [Ru(η^6^-*p*-cym)(dpa)(H_2_O)]^2+^ and free carboxylates (Figure 6). The positions of ^1^H-NMR signals of the formed complex species (e.g., at 7.89 ppm for C4–H or at 2.01 ppm for C20–H for the hydrolyzed form of the complex **4**) were consistent with those of the hydrolyzed halogenido complexes (see above), which strongly suggested the same composition of the complex species (most likely aqua complexes [Ru(η^6^-*p*-cym)(dpa)(H_2_O)]^2+^) formed by the hydrolysis of all the complexes **1**–**5**. Further, the signals of the released carboxylato ligands appeared in the ^1^H-NMR spectra with the δ values well-correlating with free carboxylate anions (Figure 6). As for the valproato complex **4**, the triplet of the terminal methyl groups (i.e., C25–H) of the VP ligand showed at 0.62 ppm in the ^1^H-NMR spectra recorded on the fresh 10% MeOD-*d*_4_/90% D_2_O solutions. Together with this signal, another triplet was detected at 0.78 ppm, whose integral intensity increased in time and whose position correlated with that of free valproate(1–) anion (0.78 ppm in the same mixture of solvents), thus this signal can be unambiguously assigned to the released VP ligand. Regarding the 4-phenylbutyrato complex **5**, the ^1^H-NMR multiplets of the aliphatic hydrogens of the PB ligand appeared at 1.65, 2.17 and 2.25 ppm, for the fresh 10% MeOD-*d*_4_/90% D_2_O solutions of complex **5** and its mixture with GSH. A hydrolytic release of the PB ligand led to the changes of these positions to 1.77, 2.10 and 2.54 ppm, which correlated well with the positions of these signals detected in the spectrum of free 4-phenylbutyrate (the experiment performed with PBA in 10% MeOD-*d*_4_/90% D_2_O).

**Figure 7:**
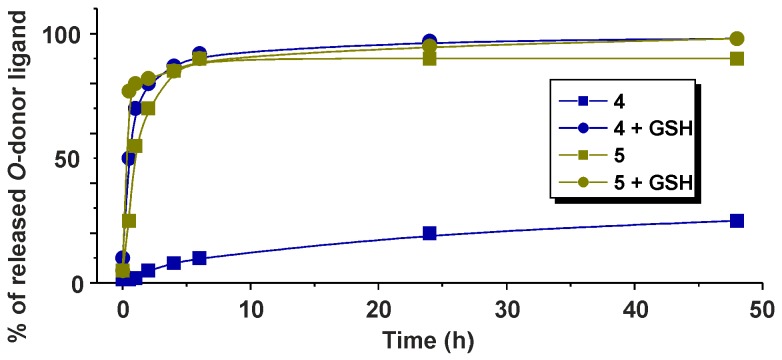
Progress of hydrolysis of the carboxylato complexes **4** (blue) and **5** (dark yellow), with (circles) or without (squares) the addition of 2 molar equivalents of the reduced glutathione (GSH). Complexes were dissolved in 10% MeOD-*d*_4_/90% D_2_O and the ^1^H-NMR spectra were recorded on the fresh solutions (0 h) and after 0.5, 1, 2, 4, 6, 24 and 48 h of standing at ambient temperature.

Interestingly, the carboxylato complexes **4** and **5** differed markedly one from another, in connection with the hydrolysis process. It has been observed that ca. 90% of the PB-containing complex **5** hydrolyzed after 48 h of standing at ambient temperature, while the valproato complex **4** was more stable and only ca. 25% hydrolyzed (Figure 7). Remarkably, although no signs of interactions of the carboxylato complexes (or their hydrolysates) with GSH were observed in the acquired ^1^H-NMR spectra, the presence of GSH in the mixtures with complexes **4** or **5** enhanced the release of the appropriate carboxylato ligand up to ca. 98% for both complexes **4** and **5** after 48 h of standing at ambient temperature (Figure 7).

### 2.4. In Vitro Cytotoxicity

In this work, the structure of the recently reported complex [Ru(η^6^-*p*-cym)(dpa)Cl]PF_6_ (**1** in this work) [10,11] was modified by the replacement of the chlorido ligand by either bromido (**2**) or iodido (**3**) ones (known approach [3,17]), or by valproato (**4**) or 4-phenylbutyrato (**5**) ones (innovative approach). Complex **1**, recently reported as cytotoxic against the MCF-7 cells (IC_50_ = 40.8 µM) [11], did not show any cytotoxicity in this work, up to the highest tested concentration (IC_50_ > 100 µM) on the A2780 cells. Similarly, the different sensitivity of various types of cancer cells was reported for the Ru(II) half-sandwich complex [Ru(η^6^-*p*-cym)(L^6^)Cl]PF_6_ containing 2-pyridylpropylimine (L^6^), which was inactive against the A2780 cells (IC_50_ > 200 µM) [33], but showed moderate activity at the MG63 human osteosarcoma cells (IC_50_ = 88.5 µM) [34]. The replacement of the chlorido ligand of the inactive complex **1** by the bromido (for **2**) or iodido (for **3**) ones did not provide potent Ru(II) complexes. Recently, similar half-sandwich Ru(II) bromido [17] and iodido [3] complexes were described in the literature as in vitro cytotoxic against the A2780 cells, with even higher activity against some of the used cell lines as compared with their chlorido analogues. In particular, complex [Ru(η^6^-*p*-cym)(L^7^)Br] is more potent (IC_50_ = 2.9 µM) than its chlorido analog [Ru(η^6^-*p*-cym)(L^7^)Cl] (IC_50_ = 4.6 µM) against the NCI-H460 non-small cell lung carcinoma; HL^7^ = 5,7-diiodo-8-quinoline [17]. Regarding iodido complexes, cytotoxicity of the [Ru(η^6^-*p*-cym)(L^1^)Cl]PF_6_ complex against the A2780 cells (IC_50_ = 16.2 µM) was lower than for the iodido complex [Ru(η^6^-*p*-cym)(L^1^)I]PF_6_ (IC_50_ = 3.0 µM) [3]. Finally and surprisingly, no activity was detected at the used A2780 cells for the Ru(II) carboxylato complexes **4** and **5**, bearing the biologically active *O*-donor ligands themselves.

## 3. Materials and Methods

### 3.1. Materials

The chemicals (RuCl_3_∙*x*H_2_O, 2,2′-dipyridylamine, valproic acid, 4-phenylbutyric acid, sodium hydroxide, silver trifluoromethanesulfonate (silver triflate), potassium chloride, potassium bromide, potassium iodide, ammonium hexafluorophosphate, reduced glutathione (GSH)) and solvents (methanol, diethyl ether, *n*-hexane, dichloromethane, DMSO-*d*_6_, MeOD-*d*_4_, D_2_O) were supplied by VWR International (Stříbrná Skalice, Czech Republic), Sigma-Aldrich (Prague, Czech Republic), Fisher Scientific (Pardubice, Czech Republic), Litolab (Chudobín, Czech Republic) and Precious Metals Online (University of Wollongong, Wollongong, Australia).

### 3.2. Syntheses

[Ru(µ-Cl)(η^6^-*p*-cym)Cl]_2_ was prepared according to the reported synthetic procedure performed in the microwave reactor [35]. Silver valproate (Ag(VP)) and silver 4-phenylbutyrate (Ag(PB)) were obtained by the neutralization of the methanolic solutions of valproic or 4-phenylbutyric acid with the stoichiometric amount of 1 M NaOH (5 min of stirring at ambient temperature) followed by the addition of 1 molar equivalent of silver triflate (5 min of stirring at ambient temperature in the dark).

#### 3.2.1. Synthesis of [Ru(η^6^-*p*-cym)(dpa)Cl]PF_6_ (**1**)

**Figure 8:**
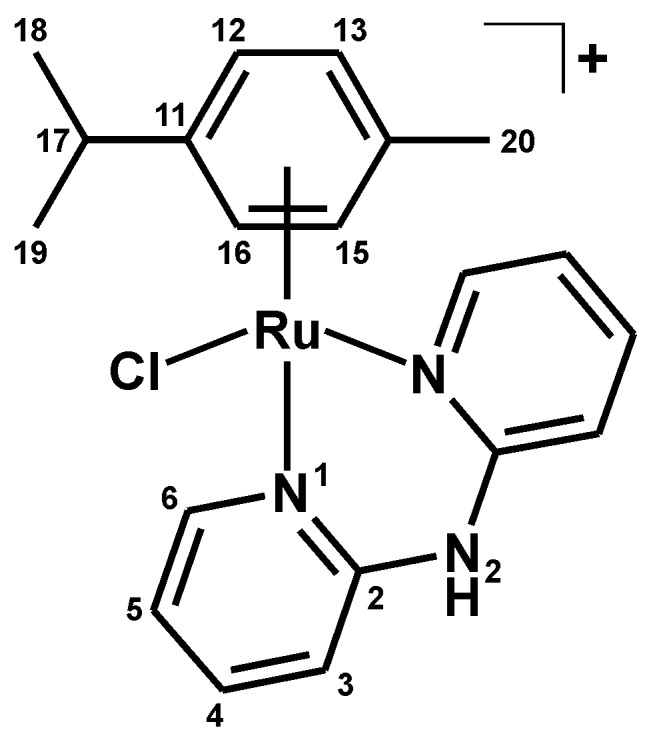
Structural formula of the complex cation in [Ru(η^6^-*p*-cym)(dpa)Cl]PF_6_ (**1**).

The mixture of [Ru(µ-Cl)(η^6^-*p*-cym)Cl]_2_ (0.5 mmol) and dpa (1.0 mmol) in methanol (15 mL) reacted in a microwave reaction system for 1 min at 100 °C, leading to the color change from orange-red to yellow. The obtained solution of [Ru(η^6^-*p*-cym)(dpa)Cl]Cl (1*) was cooled to the ambient temperature and NH_4_PF_6_ (2.5 mmol) was added. The yellow precipitate of the complex **1** (Figure 8) was collected by filtration, washed with methanol (1 × 2 mL) and diethyl ether (3 × 2 mL), and dried under vacuum in desiccator. A yield was ca. 85% (related to the starting Ru(II) dimer). Anal. Calcd. for RuC_20_H_23_N_3_ClPF_6_: C, 40.93; H, 3.95; N, 7.16%; found: C, 40.86; H, 3.97; N, 6.95%. ^1^H-NMR (DMSO-*d*_6_, ppm): δ 10.87 (s, N2–H, 1H), 8.55 (d, *J* = 5.5 Hz, C6–H, 2H), 7.97 (t, *J* = 7.8 Hz, C4–H, 2H), 7.22 (m, C3–H, C5–H, 4H), 5.73 (d, *J* = 6.4 Hz, C13–H, C15–H, 2H), 5.56 (d, *J* = 6.4 Hz, C12–H, C16–H, 2H), 2.50 (C17–H), 1.84 (s, C20–H, 3H), 1.13 (d, *J* = 7.3 Hz, C18–H, C19–H, 6H). ^1^H-NMR (MeOD-*d*_4_, ppm): δ 8.63 (d, *J* = 5.5 Hz, C6–H, 2H), 7.93 (t, *J* = 7.6 Hz, C4–H, 2H), 7.21 (m, C3–H, C5–H, 4H), 5.67 (d, *J* = 6.2 Hz, C13–H, C15–H, 2H), 5.57 (d, *J* = 6.2 Hz, C12–H, C16–H, 2H), 2.62 (sep, *J* = 6.9 Hz, C17–H), 2.10 (s, C20–H, 3H), 1.22 (d, *J* = 6.9 Hz, C18–H, C19–H, 6H). ^13^C-NMR (DMSO-*d*_6_, ppm): δ 154.6, 152.4, 140.5, 119.2, 113.8, 105.4, 99.7, 85.1, 83.8, 30.3, 21.7, 17.7. ESI + MS (methanol, *m*/*z*): 442.0 (calc. 442.0; 50%; [Ru(*p*-cym)(dpa)Cl]^+^), 406.2 (calc. 406.1; 100%; [Ru(*p*-cym)(dpa–H)]^+^). FTIR (*ν*_ATR_/cm^−1^): 448w, 463w, 533m, 641w, 677w, 762s, 827vs, 878m, 966w, 1026w, 1058w, 1092w, 1125w, 1161m, 1233w, 1279w, 1341m, 1376w, 1392w, 1437s, 1464s, 1491w, 1523w, 1582m, 1625s, 2961m, 2980m, 3025m, 3088m, 3134m, 3192m, 3223m, 3262m.

#### 3.2.2. Synthesis of [Ru(η^6^-*p*-cym)(dpa)Br]PF_6_ (**2**)

**Figure 9:**
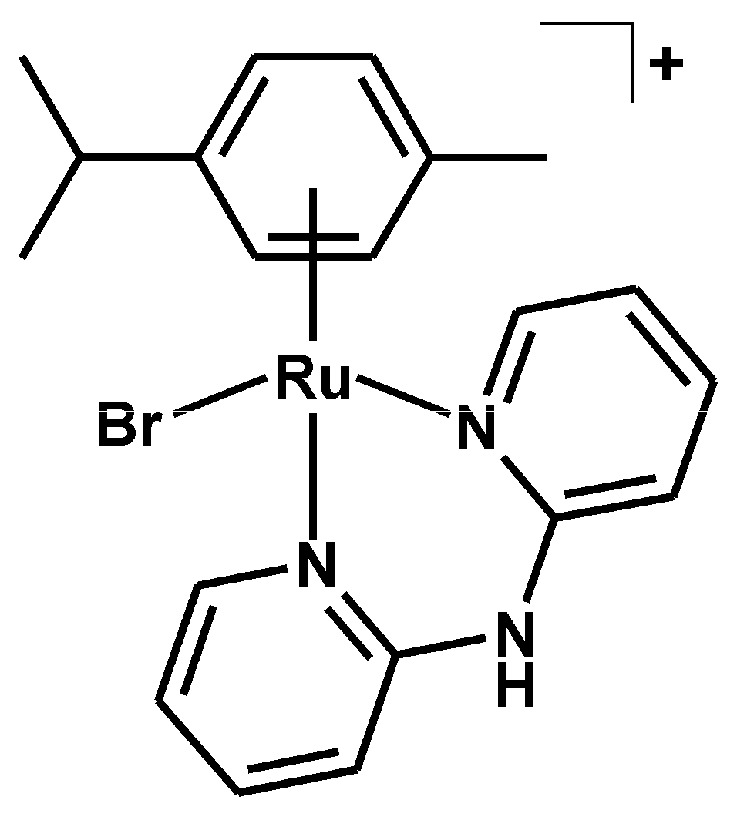
Structural formula of the complex cation in [Ru(η^6^-*p*-cym)(dpa)Br]PF_6_ (**2**).

[Ru(η^6^-*p*-cym)(dpa)Cl]Cl (1*; 0.1 mmol), prepared as described above for **1** and further used in situ, was left to interact with silver triflate (0.2 mmol) at ambient temperature in the dark (under aluminum foil) for 1 h. The white precipitate of AgCl was filtered off and 0.25 mmol of KBr was added to the obtained pure yellow filtrate. NH_4_PF_6_ (0.5 mmol) was added to the obtained orange solution after 3 h of stirring at ambient temperature. The light orange precipitate of the complex **2** (Figure 9) was collected, washed with methanol (1 × 1 mL) and diethyl ether (3 × 2 mL) and dried under vacuum in desiccator. A yield was ca. 70% (related to the starting Ru(II) dimer). Anal. Calcd. for RuC_20_H_23_N_3_BrPF_6_: C, 38.05; H, 3.67; N, 6.66%; found: C, 37.82; H, 3.67; N, 6.35%. ^1^H-NMR (DMSO-*d*_6_, ppm): δ 10.88 (s, N2–H, 1H), 8.62 (d, *J* = 5.5 Hz, C6–H, 2H), 7.96 (t, *J* = 7.3 Hz, C4–H, 2H), 7.20 (m, C3–H, C5–H, 4H), 5.75 (d, *J* = 6.4 Hz, C13–H, C15–H, 2H), 5.68 (d, *J* = 6.4 Hz, C12–H, C16–H, 2H), 2.50 (C17–H), 2.06 (s, C20–H, 3H), 1.13 (d, *J* = 6.4 Hz, C18–H, C19–H, 6H). ^13^C-NMR (DMSO-*d*_6_, ppm): δ 155.7, 152.4, 140.5, 119.2, 113.8, 106.1, 99.5, 85.5, 83.7, 30.5, 21.7, 18.1. ESI + MS (methanol, *m*/*z*): 488.0 (calc. 488.0; 100%; [Ru(*p*-cym)(dpa)Br]^+^), 406.2 (calc. 406.1; 60%; [Ru(*p*-cym)(dpa–H)]^+^). FTIR (*ν*_ATR_/cm^−1^): 464w, 532m, 555s, 639w, 677w, 762s, 828vs, 877m, 1024w, 1057w, 1161m, 1233w, 1342m, 1376w, 1392w, 1436s, 1465s, 1582m, 1625m, 2978w, 3023w, 3088m, 3133m, 1392m, 3223m, 3260w.

#### 3.2.3. Synthesis of [Ru(η^6^-*p*-cym)(dpa)I]PF_6_ (**3**)

**Figure 10:**
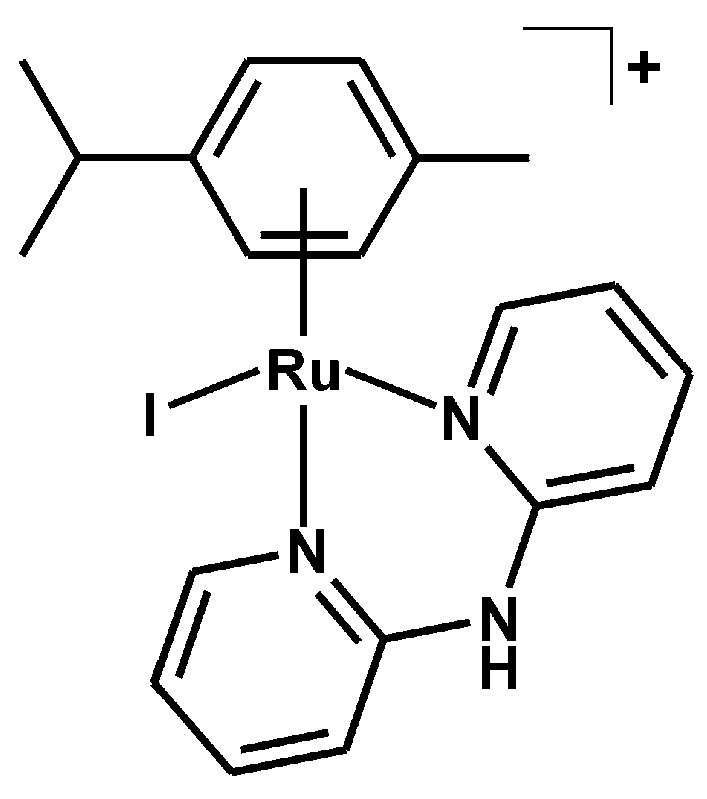
Structural formula of the complex cation in [Ru(η^6^-*p*-cym)(dpa)I]PF_6_ (**3**).

The complex **3** (Figure 10) was prepared as described for the complex **2**, with KI used instead of KBr. A yield of orange product was ca. 70% (related to the starting Ru(II) dimer). Anal. Calcd. for RuC_20_H_23_N_3_IPF_6_: C, 35.41; H, 3.42; N, 6.19%; found: C, 35.42; H, 3.60; N, 5.99%. ^1^H-NMR (DMSO-*d*_6_, ppm): δ 10.89 (s, N2–H, 1H), 8.71 (br, C6–H, 2H), 7.94 (br, C4–H, 2H), 7.18 (m, C3–H, C5–H, 4H), 5.75 (m, C12–H, C13–H, C15–H, C16–H, 4H), 2.50 (C17–H), 2.17 (s, C20–H, 3H), 1.12 (br, C18–H, C19–H, 6H). ^13^C-NMR (DMSO-*d*_6_, ppm): δ 157.3, 152.3, 140.4, 119.1, 113.9, 107.4, 99.3, 86.0, 83.6, 30.8, 21.8, 18.9. ESI + MS (methanol, *m*/*z*): 534.0 (calc. 534.0; 100%; [Ru(*p*-cym)(dpa)I]^+^), 406.2 (calc. 406.1; 40%; [Ru(*p*-cym)(dpa–H)]^+^). FTIR (*ν*_ATR_/cm^−1^): 452w, 462w, 530w, 555s, 636w, 672w, 745m, 831vs, 880m, 1033w, 1059w, 1122w, 1160m, 1233w, 1348m, 1382w, 1433m, 1468s, 1523w, 1584m, 1626m, 2933w, 2967w, 3045w, 3092w, 3224w, 3253w, 3371s.

Recrystallization of the complex **3** from the dichloromethane/*n*-hexane mixture of solvents provided the crystals suitable for a single-crystal X-ray analysis.

#### 3.2.4. Synthesis of [Ru(η^6^-*p*-cym)(dpa)(VP)]PF_6_ (**4**)

**Figure 11:**
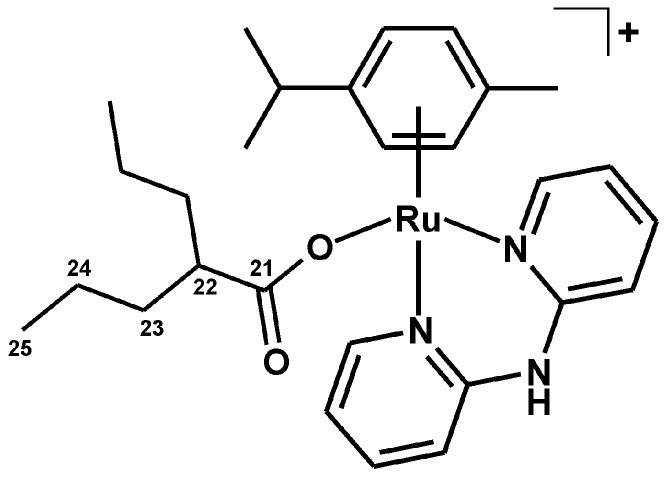
Structural formula of the complex cation in [Ru(η^6^-*p*-cym)(dpa)(VP)]PF_6_ (**4**); VP = valproato(1–).

The complex **1** (0.1 mmol) was dissolved in methanol (50 mL) and 0.35 mmol of Ag(VP) was added. The mixture of the formed AgCl and unreacted Ag(VP) was filtered off after the overnight stirring in the dark (under aluminum foil) at ambient temperature, and NH_4_PF_6_ was added (0.5 mmol). Then the reaction mixture was evaporated (using the nitrogen gas) until the light yellow solid precipitated. The product (Figure 11) was collected by filtration, washed with methanol (1 × 1 mL) and diethyl ether (3 × 2 mL) and dried under vacuum in desiccator. The yield was ca. 65% (related to the starting complex **1**). Anal. Calcd. for RuC_28_H_38_N_3_O_2_PF_6_: C, 48.41; H, 5.51; N, 6.05%; found: C, 48.03; H, 5.81; N, 6.11%. ^1^H-NMR (DMSO-*d*_6_, ppm): δ 10.83 (s, N2–H, 1H), 8.73 (d, *J* = 5.5 Hz, C6–H, 2H), 7.97 (t, *J* = 7.6 Hz, C4–H, 2H), 7.23 (d, *J* = 7.6 Hz, C3–H, C5–H, 2H), 5.84 (d, *J* = 5.9 Hz, C13–H, C15–H, 2H), 5.79 (d, *J* = 5.9 Hz, C12–H, C16–H, 2H), 2.40 (sep, *J* = 6.8 Hz, C17–H, 1H), 2.04 (m, C22–H, 1H), 1.93 (s, C20–H, 3H), 1.25 (m, C23–H, C23′–H, 4H), 1.08 (d, *J* = 6.2, C18–H, C19–H, 6H), 0.97 (m, C24–H, C24′–H, 4H), 0.62 (t, *J* = 7.2 Hz, C25–H, C25′–H, 6H). ^13^C-NMR (DMSO-*d*_6_, ppm): δ 181.1, 153.7, 152.0, 140.3, 128.6, 125.9, 118.7, 113.7, 103.8, 99.4, 83.7, 83.3, 46.7, 35.1, 30.0, 23.8, 21.6, 19.9, 17.3, 13.7. ESI + MS (methanol, *m*/*z*): 549.8 (calc. 550.2; 15%; [Ru(*p*-cym)(dpa)(VP)]^+^), 406.2 (calc. 406.1; 100%; [Ru(*p*-cym)(dpa–H)]^+^). FTIR (*ν*_ATR_/cm^−1^): 467w, 552w, 614w, 699w, 771m, 833vs, 881w, 1006w, 1029w, 1058w, 1165w, 1229w, 1242w, 1306w, 1362m, 1384m, 1437m, 1475s, 1533m, 1579s, 1619m, 1644m, 2872m, 2935s, 2957s, 3081w, 3192w, 3241w, 3299w.

#### 3.2.5. Synthesis of [Ru(η^6^-*p*-cym)(dpa)(PB)]PF_6_ (**5**)

**Figure 12:**
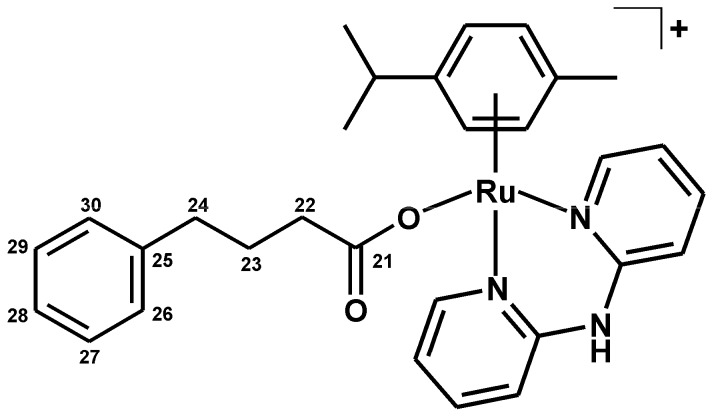
Structural formula of the complex cation in [Ru(η^6^-*p*-cym)(dpa)(PB)]PF_6_ (**5**); PB = 4-phenylbutyrato(1–).

The complex **5** (Figure 12) was prepared as described for complex **4**, with Ag(PB) used instead of Ag(VP). A yield was ca. 60% (related to the starting complex **1**). Anal. Calcd. for RuC_30_H_34_N_3_O_2_PF_6_: C, 50.42; H, 4.80; N, 5.88%; found: C, 50.05; H, 4.80; N, 5.48%. ^1^H-NMR (DMSO-*d*_6_, ppm): δ 8.77 (d, *J* = 5.1 Hz, C6–H, 2H), 7.95 (t, *J* = 7.2 Hz, C4–H, 2H), 7.20 (m, C3–H, C5–H, 2H), 7.11 (m, C27–H, C28–H, C29–H, 3H), 6.92 (m, C26–H, C30–H, 2H), 5.86 (d, *J* = 6.3 Hz, C13–H, C15–H, 2H), 5.75 (d, *J* = 6.3 Hz, C12–H, C16–H, 2H), 2.40 (sep, *J* = 6.7 Hz, C17–H, 1H), 2.31 (t, *J* = 7.4 Hz, C22–H, 2H), 2.07 (t, *J* = 7.0 Hz, C24–H, 2H), 1.89 (s, C20–H, 3H), 1.67 (qui, *J* = 7.3 Hz, C23–H, 2H), 1.09 (d, *J* = 6.7 Hz, C18–H, C19–H, 6H). ^13^C-NMR (DMSO-*d*_6_, ppm): δ 179.0, 153.8, 152.3, 142.0, 140.5, 128.1, 125.6, 119.0, 114.1, 103.8, 99.6, 84.0, 83.7, 36.4, 34.6, 28.1, 24.0, 21.8, 17.5. ESI + MS (methanol, *m*/*z*): 569.8 (calc. 570.2; 10%; [Ru(*p*-cym)(dpa)(PB)]^+^), 406.2 (calc. 406.1; 100%; [Ru(*p*-cym)(dpa)]^+^). FTIR (*ν*_ATR_/cm^−1^): 439w, 494w, 538w, 555s, 648w, 663w, 701m, 759m, 781m, 830vs, 874w, 1029w, 1063w, 1082w, 1158w, 1224m, 1303m, 1349m, 1387s, 1413w, 1434m, 1467s, 1490w, 1530w, 1571m, 1615w, 1639w, 2867m, 2930m, 2967m, 3005w, 3065w, 3183w, 3184w, 3232w, 3287w.

### 3.3. Methods

^1^H, ^13^C, ^1^H–^1^H gs-COSY, ^1^H–^13^C gs-HMQC and ^1^H–^13^C gs-HMBC spectra were acquired for DMSO-*d_6_* solutions at 298 K on a JEOL JNM-ECA 600II device at 600.00 MHz (^1^H) and 150.86 MHz (^13^C); gs = gradient selected, COSY = correlation spectroscopy, HMQC = heteronuclear multiple quantum coherence, HMBC = heteronuclear multiple bond coherence. The spectra were calibrated against the residual signals of the used solvent at 2.50 ppm (^1^H-NMR) and 39.52 ppm (^13^C-NMR) [36]. The splitting of the ^1^H-NMR signals is defined as s = singlet, d = doublet, t = triplet, qui = quintet, dt = doublet of triplets, br = broad band, m = multiplet. Electrospray ionization (ESI) mass spectra of the methanol solutions were obtained of on a LCQ Fleet Ion Trap mass spectrometer (Thermo Scientific; Qual Browser software, version 2.0.7; Waltham, MA, USA) in the positive ionization mode (ESI+). Elemental analysis (C, H, N) was performed using a Flash 2000 CHNS Elemental Analyzer (Thermo Scientific). FTIR spectra were recorded using Nexus 670 FT-IR (Thermo Nicolet) on an ATR diamond plate between 400 and 4000 cm^−1^. RP-HPLC experiments were performed using UHPLC-MS (Dionex/Thermo Scientific) mass spectrometer and an ReproSil-Pur Basic C18, 5 μm pore size, 200 × 4.6 mm. Mobile phase used was H_2_O 0.1% Htfa/MeCN at gradients of t = 0 min 10% B, t = 30 min 80% B, t = 40 min 80% B, t = 41 min 10% B, and t = 55 min 10% B over a 55 min period. Flow rate was 1 mL·min^−1^, and the detection wavelength was set at 254 nm. H_2_O and acetonitrile (MeCN) of HPLC grade were used for the RP-HPLC experiments with an addition of trifluoroacetic acid (Htfa).

A Monowave 300 (Anton PaarGmbH, Graz, Austria) microwave reactor was used for the synthesis of the starting dimeric Ru(II) compound as well as for the chlorido complexes 1* and **1** (30 mL microwave vials equipped with magnetic stirring bars).

A suitable single crystal of [Ru(η*^6^-p*-cym)(dpa)I]PF_6_ (**3**) was selected and placed on an D8 QUEST monocrystal diffractometer (Bruker, Billerica, MA, USA) with PHOTON 100 CMOS detector, using the Mo–Kα radiation (λ = 0.71075 Å). The APEX3 software package was used for data collection and reduction [37]. The structures were solved using a direct method and refined using the Bruker SHELXTL Software Package (Bruker) [38]. X-ray crystallographic data have been deposited in the Cambridge Crystallographic Data Centre (Cambridge, United Kingdom) under the accession number CCDC 1515628. The graphics were drawn and additional structural calculations were performed by DIAMOND (Version 4.0.3.; Crystal Impact GbR, Bonn, Germany) [39] and Mercury [40] software (Version 3.0; Cambridge Crystallographic Data Centre, Cambridge, United Kingdom).

### 3.4. ^1^H-NMR Studies of Aqueous Chemistry and Interactions with GSH

Complexes **1**–**5** were dissolved in 60 µL of MeOD-*d*_4_ and diluted with 540 µL of D_2_O to get the 1 mM solutions. The ^1^H-NMR spectra were recorded immediately after the preparation of the samples (0 h) and after 0.5, 1, 2, 4, 6, 24 and 48 h of standing at ambient temperature. After that, 5 molar equivalent of KCl (for **1**), KBr (for **2**) or KI (for **3**) were added to the solutions and the ^1^H-NMR spectroscopy was carried out on the fresh solutions (0 h) and after 1, 6 and 24 h of standing at ambient temperature. Similar experiments were performed with an addition of GSH, as follows: **1**–**5** (amounts necessary for the final concentration of 1 mM) were dissolved in 60 µL of MeOD-*d*_4_ and 2 molar equivalent of GSH dissolved in 540 µL of D_2_O were added. The ^1^H-NMR spectra were acquired right after the preparation of the solutions (0 h) and after 0.5, 1, 2, 4, 6 and 24 h of standing at ambient temperature. The obtained ^1^H-NMR spectra were calibrated against the residual signal of D_2_O found at 4.85 ppm.

### 3.5. Cell Culture and In Vitro Cytotoxicity

The A2780 human ovarian carcinoma cells, purchased from the European Collection of Cell Cultures (ECACC), were cultured in RPMI-1640 medium supplemented with 10% of fetal calf serum, 1% of 2 mM glutamine and 1% penicillin/streptomycin, according to the ECACC instructions. The cells were grown at 37 °C and 5% CO_2_ in a humidified incubator as adherent monolayers.

The cultured A2780 cells were seated in the 96-well culture plates and pre-incubated in drug-free media at 37 °C for 24 h. After that, the cells were treated with the 0.01–100.0 μM solutions of complexes **1**–**5** and *cisplatin* (prepared by appropriate dilution of the fresh 100 mM stock solutions of the tested substances dissolved in DMF) for 24 h (exposure time) at 37 °C. Then, the solutions containing the tested compounds were removed and the cells were washed with drug-free medium and kept under drug-free medium for the next 72 h (recovery time). In parallel, the cells were treated with vehicle (0.1% DMF, *v/v*) and Triton X-100 (1%, *v/v*) to assess the minimal (100% viability) and maximal (0% viability) cell damage, respectively.

The in vitro cytotoxicity was assessed using an MTT assay and evaluated spectrophotometrically at 540 nm (Tecan Group Ltd., Männedorf, Switzerland). The cytotoxicity data were received from three independent experiments (each conducted in triplicate) using the cells from three different passages. The resulting IC_50_ values (µM) were calculated from viability curves and the results are presented as arithmetic mean ± SD.

## 4. Conclusions

A series of the half-sandwich ruthenium(II) complexes of the general formula [Ru(η^6^-*p*-cym)(dpa)X]PF_6_ (**1**–**5**), showing a pseudo-octahedral piano-stool geometry, has been prepared and fully characterized. The halogenido complexes **1**–**3** contain the chlorido, bromido, and iodido ligand, respectively. For the first time, we report on the organometallic ruthenium(II) complexes **4** and **5** containing the monodentate *O*-donor valproato (VP), and 4-phneylbutyrato (PB) ligand, respectively. The obtained results of the solution behavior in a water-containing solution clearly proved a release of the halogenido (for **1**–**3**) and carboxylato (for **4** and **5**) ligands used. The rate of release of the ligands is increased in the presence of the reduced glutathione. The complexes **1**–**5** did not show any cytotoxic effect in vitro up to the highest tested concentration (IC_50_ > 100 μM) against the A2780 human ovarian carcinoma cells.

## Figures and Tables

**Table 1:** ^1^H-NMR coordination shifts (Δδ = δ_complex_ − δ_ligand_; ppm) for the dpa ligand of **1**–**5**.

Complex	N2–H	C3–H	C4–H	C5–H	C6–H
**1**	1.21	−0.52	0.34	0.38	0.34
**2**	1.22	−0.54	0.33	0.36	0.41
**3**	1.24	−0.56	0.31	0.34	0.50
**4**	1.17	−0.51	0.34	0.39	0.52
**5**	–	−0.54	0.32	0.36	0.56

**Table 2:** Crystal data and structure refinement for [Ru(η^6^-*p*-cym)(dpa)I]PF_6_ (**3**).

Empirical Formula	C_20_H_23_F_6_IN_3_PRu
Formula weight	678.35
Temperature (K)	120(2)
Wavelength (Å)	0.71073
Crystal system	Triclinic, *P*-1
Unit cell dimensions	
*a* (Å)	8.708(3)
*b* (Å)	10.275(4)
*c* (Å)	13.349(5)
*α* (°)	93.15(2)
*β* (°)	105.564(15)
*γ* (°)	94.245(17)
*V* (Å^3^)	1143.9(8)
*Z*, *D*_calc_ (g·cm^−3^)	2, 1.969
Absorption coefficient (mm^−1^)	2.167
Crystal size (mm)	0.160 × 0.100 × 0.100
F (000)	660
θ range for data collection (°)	2.438 to 24.999
Index ranges (*h*, *k*, *l*)	−10 ≤ *h* ≤ 10
	−12 ≤ *k* ≤ 12
	−15 ≤ *l* ≤ 15
Reflections collected	26913
Independent reflections	4035 [*R*(int) = 0.0393]
Data/restraints/parameters	4035/1/295
Goodness–of–fit on F^2^	1.052
Final *R* indices [*I* > 2σ(*I*)]	*R*_1_ = 0.0202, w*R*_2_ = 0.0499
*R* indices (all data)	*R*_1_ = 0.0241, w*R*_2_ = 0.0516
Largest peak and hole (e·Å^−3^)	0.585 and −0.871

**Table 3:** Comparison of the selected bond lengths (Å) and angles (°) of the complexes [Ru(η^6^-*p*-cym)(dpa)Cl]PF_6_ (**1**) ^1^ and [Ru(η^6^-*p*-cym)(dpa)I]PF_6_ (**3**).

Parameter ^2^	1	3
Ru1–X1	2.4148(7)	2.7277(10)
Ru1–N1	2.107(2)	2.101(2)
Ru1–N1A	2.096(2)	2.112(2)
Ru1–*Cg*	1.6817(2)	1.6895(6)
Ru1–C11	2.206(2)	2.239(3)
Ru1–C12	2.202(2)	2.197(3)
Ru1–C13	2.215(3)	2.197(3)
Ru1–C14	2.237(2)	2.210(3)
Ru1–C15	2.203(2)	2.184(3)
Ru1–C16	2.169(3)	2.212(3)
X1–Ru1–N1	87.04(7)	87.52(6)
X1–Ru1–N1A	87.26(6)	88.46(6)
X1–Ru1–*Cg*	127.32(6)	127.54(2)
N1–Ru1–N1A	82.30(8)	84.47(8)
N1–Ru1–*Cg*	128.72(6)	127.27(6)
N1A–Ru1–*Cg*	127.32(6)	127.59(6)

^1^ Crystallographic data have been taken from the literature [10] (CSD refcode: IKEKUF); ^2^ X = Cl (**1**) or I (**3**); *Cg* = the centroid of the *p*-cymene aromatic ring.

## Supplementary Materials

Supplementary materials can be accessed at: http://www.mdpi.com/1420-3049/21/12/1725/s1.
