# Supplementary material for: Half-Sandwich Ru(II) Halogenido, Valproato and 4-Phenylbutyrato Complexes Containing 2,2′-Dipyridylamine: Synthesis, Characterization, Solution Chemistry and In Vitro Cytotoxicity
Source: Molecules. 2016 Dec 15;21(12):1725. doi: 10.3390/molecules21121725 (PMC6274116; doi:10.3390/molecules21121725)
Supplement: Supplementary file 1 [file molecules-21-01725-s001.pdf]

Supplementary Materials: Half-Sandwich Ru(II) Halogenido, Valproato and 4-Phenylbutyrato Complexes Containing 2,2′-Dipyridylamine: Synthesis, Characterization, Solution Chemistry and In Vitro Cytotoxicity

Pavel Štarha, Zdeněk Trávníček, Radka Křikavová and Zdeněk Dvořák

Table S1. Parameters of selected non-covalent contacts (Å, °) detected in the crystal structure of [Ru(η^6-*p*-cym)(dpa)I]PF_6 (**3**).

Contact	*d*(D–H) (Å)	*d*(H···A) (Å)	*d*(D–H···A) (Å)	<(D–H···A) (°)
N2–H2A···F4 ^{i}	0.85(2)	2.40(3)	3.096(3)	140(3)
N2–H2A···F6 ^{i}	0.85(2)	2.14(3)	2.953(3)	161(3)
C3–H3A···F6 ^{i}	0.95	2.60	3.351(3)	136.3(2)
C4–H4A···F1 ^{ii}	0.95	2.53	3.210(4)	128.7(2)
C4–H4A···F5 ^{ii}	0.95	2.61	3.557(4)	173.8(2)
C15–H15A···F3 ^{iii}	0.95	2.47	3.069(4)	121.1(2)
C16–H16A···F3 ^{iii}	0.95	2.48	3.068(3)	120.44(14)

Symmetry codes: (i) x − 1, y, z; (ii) 1 − x, −y, 1 − z; (iii) 2 − x, 1 − y, 1 − z.

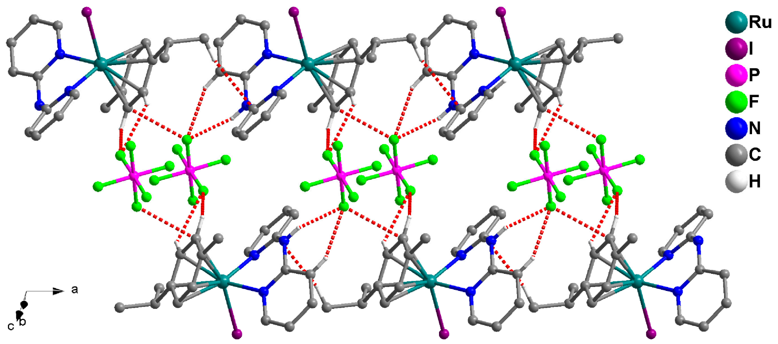

Figure S1. Part of the crystal structure of [Ru(η^6-*p*-cym)(dpa)I]PF_6 (**3**) with non-covalent contacts depicted by red dashed lines. The hydrogen atoms not involved into the non-covalent contacts have been omitted for clarity.

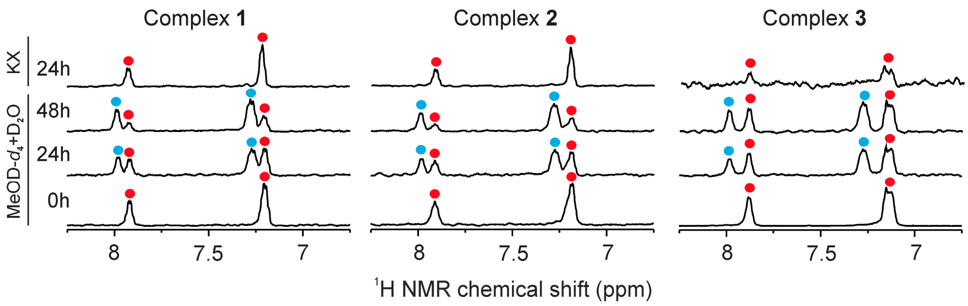

Figure S2. ^{1}H-NMR spectra acquired on 10% MeOD-d_4/90% D_2O solutions for the halogenido complexes **1**–**3** in different time points (0 h, 24 h and 48 h) and after the addition of the appropriate potassium halogenide (KX = KCl (for **1**), KBr (for **2**) or KI (for **3**)). The signals of the initial complex are labelled by the red spheres, while the signals of hydrolysates are labelled by the blue ones.
